# Interfacial engineering of renewable metal organic framework derived honeycomb-like nanoporous aluminum hydroxide with tunable porosity†
†Electronic supplementary information (ESI) available. See DOI: 10.1039/c6sc05695d



**DOI:** 10.1039/c6sc05695d

**Published:** 2017-02-28

**Authors:** Ye-Tang Pan, Lu Zhang, Xiaomin Zhao, De-Yi Wang

**Affiliations:** a IMDEA Materials Institute , C/Eric Kandel, 2 , 28906 Getafe , Madrid , Spain . Email: deyi.wang@imdea.org

## Abstract

Novel honeycomb-like mesoporous aluminum hydroxide (pATH) was synthesized *via* a facile one-step reaction by employing ZIF-8 as a template. This self-decomposing template was removed automatically under acidic conditions without the need for any tedious or hazardous procedures. Meanwhile, the pore size of pATH was easily modulated by tuning the dimensions of the ZIF-8 polyhedrons. Of paramount importance was the fact that the dissolved ZIF-8 in solution was regenerated upon deprotonation of the ligand under mild alkali conditions, and was reused in the preparation of pATH, thus forming a delicate synthesis cycle. The renewable template conferred cost-effective and sustainable features to the as-synthesized product. As a proof-of-concept application, the fascinating nanoporous structure enabled pATH to load more phosphorous-containing flame retardant and endowed better interaction with epoxy resin over that of commercial aluminum hydroxide. The limiting oxygen index, UL-94 vertical burning test and cone calorimeter test showed that the results of epoxy with the modified pATH rivalled those of epoxy with two times the loading amount of the commercial counterpart, while the former presented better mechanical properties. The proposed “amorphous replica method” used in this work will advance the potential for launching a vast area of research and technology development for the preparation of porous metal hydroxides for use in practical applications.

## Abstract 2

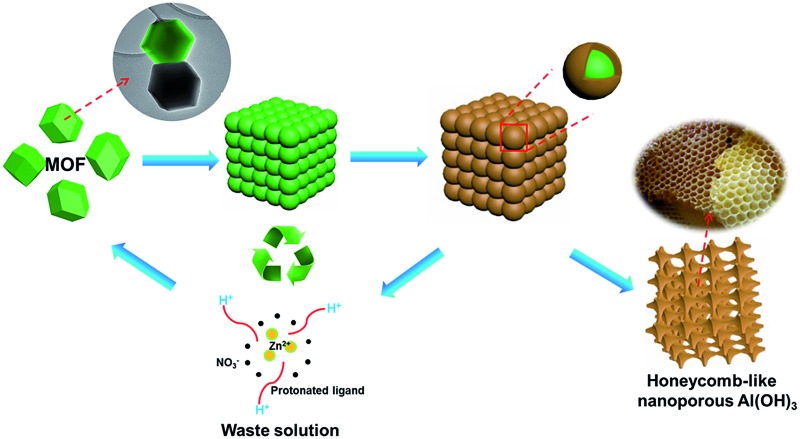
Novel honeycomb-like mesoporous aluminum hydroxide (pATH) was synthesized *via* a facile one-step reaction by employing ZIF-8 as a template.

## 1. Introduction

Nanoporous materials have prompted a surge of interest in various applications because of their fascinating, remarkable and useful properties.^1–4^ Synthetic methods for preparing porous materials are roughly categorized into two general types, which are recognized as hard-template and soft-template methods.^5^ The hard template method, which mainly uses siliceous materials, involves the tedious removal of the framework with a highly corrosive solution, which is dangerous during operation, and is also not applicable in the preparation of materials that are sensitive to acid or alkali.^6^ Recently, Yang *et al.* used colloidal silica as a hard template to synthesize mesoporous hematite crystals. The removal of the template was conducted using NH_4_HF_2_, which is a highly corrosive and toxic substance.^7^ In the case of the soft template method, the use of various structural directing agents such as surfactants or block copolymers still calls for a sequential template-removal process *via* solution extraction or calcination, which are time consuming and plagued by high energy consumption. Feng *et al.* selected polystyrene-*b*-poly(ethylene oxide) block copolymer as the soft template to fabricate mesoporous polypyrrole. The template was eliminated by repeatedly washing with tetrahydrofuran, ethanol and water.^8^ Suib *et al.* used the same block copolymer to prepare MoO_3_, followed by removal of the template with calcination under air atmosphere.^9^ Until now, nearly all research regarding porous materials is bound by the use of traditional templates, even though a myriad of disadvantages, especially the removal of the template, impede practical applications. Meanwhile, it is difficult to realize pore-size controllable synthesis in many cases. Therefore, the development of a novel self-eliminating template with tunable size remains a key issue that needs to be addressed.

Nowadays, metal organic frameworks (MOFs) are extensively employed as sacrificial matrices in the construction of functional materials.^10^ To name a few, preparations of carbon materials^11–14^ and metal oxides/sulfides/selenides^15–17^ by utilizing MOFs, especially zeolitic imidazolate frameworks (ZIFs), are under broad investigation nowadays. ZIF-8, a zinc-based metal organic framework, is very sensitive to acidic conditions, and totally degrades due to protonation of the imidazole ligand by H^+^ in acidic solutions.^18,19^ In light of this unique feature, ZIF-8 may be a good candidate as a template for the engineering of nanoporous materials accompanied by the *in situ* removal of the template synchronously if the pH value of the solution is below 7.

Li *et al.* assembled a continuous and uniform layer of aluminum hydroxide (ATH) on a graphene oxide sheet due to the intrinsic properties of aluminum ions which tend to form amorphous hydroxides. After the removal of the graphene oxide by calcination, alumina nanosheets were obtained.^20^ We named this method the “amorphous replica method”. However, the application of such a method remains in its infancy, since no follow-up report has emerged to date. Inspired by this, an amorphous aluminum hydroxide layer hydrolyzed from aluminum nitrate may also form on the hydrophilic surface of ZIF-8. As we know, the hydrolysis of aluminum nitrate renders the pH value of the solution far below 7. The ZIF-8 will decompose under these acidic conditions gradually, and the aluminum hydroxide framework will be reserved, thus duplicating the shapes of the polyhedra. Ultimately, the entire disappearance of the ZIF-8 template affords amorphous nanoporous aluminum hydroxide. The pore sizes of the final product were readily modulated by preparing ZIF-8 templates with different dimensions. To the best of our knowledge, a one-step synthesis of nanoporous aluminum hydroxide (pATH) has never been reported.

Recently, there has been intense pressure to develop cost-effective flame retardants with high efficiencies in order to reduce the occurrence of fire accidents. As a proof-of-concept application of pATH, since aluminum hydroxide is a well-recognized traditional flame retardant,^21–23^ the fire performance of an epoxy composite, in the presence of the thus-obtained product coated by a phosphorous-containing flame retardant (9,10-dihydro-9-oxa-10-phosphaphenanthrene-10-oxide, designated as DOPO), was studied, which might achieve a similar efficiency to that of the commercially available counterpart, with a much lower addition amount, while maintaining the mechanical properties of the composite. Two-dimensional (2D) nanomaterials, as a family of newly emerging flame retardants, were reviewed by Wang *et al.*, and feature high specific surface areas and the physical barrier effect.^24^ Compared to these lamellar nanomaterials, nanoporous materials have abundant porosity which is conducive to the high loading of primary flame retardants and better interaction with the polymer matrix. However, the production costs for this nanoporous aluminum hydroxide compared with those of the commercial solid should also be considered. It should be noted that MOF materials are produced from specially synthesized organic ligands (such as imidazole) to realize the optimal template, and that the large-scale manufacture of metal hydroxides or oxides by sacrificing such organic ligands may be inviable from the points of view of economic and sustainability factors.^25^ Therefore, in view of potentially solving this problem, we proposed, for the first time in this work, a facile method for the recycling of ZIF-8 from wastewater after the synthesis of pATH by a mild alkali, thus decreasing the cost of the synthetic route to the minimum. The recycled ZIF-8 was reused to prepare pATH again, thus forming a delicate synthesis cycle.

Herein, a novel well-connected aluminum hydroxide, with tunable pore width and interconnected nanoporosity emanating from ZIF-8 as a template, was synthesized and is the first based on advanced interfacial structural engineering – “amorphous replica method”. The pH-sensitivity rendered the template removal facile and automatic without any tedious or dangerous processes. Interestingly, the template was recovered from wastewater and reutilized to prepare the product repeatedly, which is indicative of a cost-effective and sustainable manufacturing technique. A potential application of mesoporous aluminum hydroxide was investigated in terms of its modification by DOPO as a flame retardant for epoxy resin (EP) to compete with the commercial counterpart. The proposed method that is consistent with the previous pioneering report opens a new avenue for artificially devised template-engaged porous nano-architectures with target functionalities.

## 2. Experimental

### 2.1 Materials

Aluminum hydroxide (reagent grade, ATH), zinc acetate dehydrate [ACS reagent, ≥98%, Zn(CH_3_COO)_2_·2H_2_O], 2-methylimidazole (99%, 2-MIM), aluminum nitrate nonahydrate [≥98%, Al(NO_3_)_3_·9H_2_O], iron(iii) nitrate nonahydrate [ACS reagent, ≥98%, Fe(NO_3_)_3_·9H_2_O], magnesium nitrate hexahydrate [ACS reagent, 99%, Mg(NO_3_)_2_·6H_2_O], methanol (anhydrous, 99.8%), ethanol (absolute alcohol, without additive, ≥98%), sodium bicarbonate (ACS reagent, ≥99.7%, NaHCO_3_), sodium hydroxide (ACS reagent, ≥97%, pellets, NaOH), ammonia solution (2 M in ethanol), (3-glycidyloxypropyl) trimethoxysilane (≥98%, KH560) and triphenylphosphine (≥95%) were all provided by Sigma-Aldrich Chemical Co. Epoxy resin (Epoxydhedraz C) was provided by R&G Faserverbundwerkstoffe GmbH-Germany. Diamino diphenyl sulfone (DDS) and DOPO were supplied by TCI Chemicals Company.

### 2.2 Preparation of ZIF-8 template

0.09 g of Zn(CH_3_COO)_2_·2H_2_O and 3.36 g of 2-MIM were dissolved in 15 ml of deionized water, respectively. Next, the two solutions were mixed together rapidly followed by aging two solutions in parallel for 2 h (denoted as ZIF-8-2 h) and 5 h (denoted as ZIF-8-5 h) separately. The white-coloured precipitate was collected by centrifugation and rinsed with methanol repeatedly. The product was dried in a vacuum desiccator at room temperature.

### 2.3 Synthesis of nanoporous aluminum hydroxide

2 g of ZIF-8 (ZIF-8-2 h and ZIF-8-5 h) was added to a 50 ml ethanol–water solution (volume ratio 1 : 1) and stirred vigorously to obtain a white suspension solution. Then 6 g of Al(NO_3_)_3_·9H_2_O was added into the solution and the mixture was continuously stirred for 30 min. After gathering by centrifugation and rinsing with ethanol and deionized water many times, the product was dried in an oven at 70 °C overnight. The samples were designated as pATH (ZIF-8-2 h as template) and macro-pATH (ZIF-8-5 h as template). Meanwhile, the waste solution which used ZIF-8-2 h as the template was reserved for further usage.

### 2.4 Synthesis of iron hydroxide and magnesium hydroxide

Iron hydroxide and magnesium hydroxide were also synthesized by adopting ZIF-8-2 h as a template according to the same procedure as that used for the synthesis of nanoporous aluminum hydroxide, except for the changes of Al(NO_3_)_3_·9H_2_O to iron nitrate and magnesium nitrate, respectively.

### 2.5 Synthesis of aluminum oxide and magnesium oxide

The as-synthesized aluminum hydroxide and magnesium hydroxide samples were calcined at 500 °C for 2 h in a muffle furnace to prepare the corresponding oxides.

### 2.6 Recycling of the ZIF-8 template from the waste solution

Different kinds of alkalis (NaOH, NaHCO_3_ and ammonia solution) were used to recover ZIF-8 from the waste solution after the synthesis of pATH. 20 ml of a 0.5 M NaOH ethanol solution was slowly added to the 50 ml waste solution with stirring and a white precipitate was immediately observed. After 2 h, the sediment was collected by centrifugation, rinsed with methanol and deionized water and dried at room temperature. 5 ml NaHCO_3_ ethanol solutions with different molar ratios (0.5 M, 1 M and 2 M) were poured into the waste solution respectively and, after stirring for a few minutes, a white precipitate emerged. After reacting for 2 h, the sample was collected using the same procedure as that mentioned above. 20 ml of ammonia solution was also added to the waste solution but, after stirring for 2 h, the mixed solution remained transparent without any sediment observed.

### 2.7 Re-synthesis of nanoporous aluminum hydroxide by the recycled ZIF-8

The ZIF-8 that was recycled using 1 M NaHCO_3_ was exploited to synthesize nanoporous aluminum hydroxide again. The same recycling and re-synthesis procedure was repeated once more.

### 2.8 Modification of ATH and pATH by DOPO

First, a KH560 containing phosphorous flame retardant (KH–DOPO) was prepared with a slight modification, according to the literature.^26^ In a typical run, 4 g of silane coupling agent, 3.66 g of DOPO and 0.383 g of triphenylphosphine were charged into a 3-mouth flask and stirred at 130 °C under an inert argon atmosphere. The procedure was controlled by measuring the intensity of the FT-IR spectra at 2400 cm^–1^ for the P–H bonds. Within 7 h, the peak near this wavenumber completely vanished, thus indicating that the reaction had finished. The resultant product was collected, followed by drying at 70 °C overnight in a vacuum oven. Secondly, 5 g of ATH and pATH were added into 50 ml of deionized water in a three-necked flask respectively with vigorous stirring to obtain a suspension liquid. The suspension was heated to 80 °C and 0.6 g of DOPO–KH560 diluted by ethanol (volume ratio 1 : 1) was added to the suspension. After stirring for 10 h, the product was filtered, washed by ethanol and dried at 70 °C. The products were named ATH–DOPO and pATH–DOPO.

### 2.9 Preparation of epoxy composites

Different weight fractions of ATH–DOPO (5, 10, 15 and 20 wt%) and pATH–DOPO (5 and 10 wt%) were adopted to prepare the epoxy composites. First, the fillers were mixed with the resin by means of a triple-roll mix (EXAKT 80E) for 0.5 h. The mixture was transferred onto a magnetic stirring apparatus and the temperature was increased to 130 °C. Under vigorous stirring, the curing agent DDS was blended into the mixture and then stirred until a homogenous solution was obtained. The bubbles were removed by leaving the solution in a vacuum oven at 100 °C for 20 min, followed by casting the solution into pre-heated polytetrafluoroethylene moulds immediately. The cure process was carried out at 160 °C for 1 h, 180 °C for 2 h and 200 °C for 2 h. After cooling down to normal temperature, the epoxy composites were obtained.

### 2.10 Measurements

X-ray diffraction (XRD) patterns were recorded by a Philip X′ Pert PRO diffractometer using Cu Kα radiation. Fourier transform infrared spectroscopy (FT-IR) spectra were obtained using a Nicolet iS50 spectrometer over a wavenumber range of 4000 to 400 cm^–1^. Nitrogen adsorption and desorption isotherms at 77 K were obtained using a Micromeritics instrument (ASAP 2020). The specific surface area and the pore size distribution were computed using the Brauner–Emmett–Teller (BET) equation and the non-local density functional theory (NLDFT) model, respectively. Transmission electron microscopy analysis (TEM) images were taken using an FE6 S/TEM microscope (Talos F200X, FEI) at 80 kV. X-ray photoelectron spectroscopy (XPS) was carried out using an Organic Molecular Beam Epitaxy (OMBE) system and the Al Kα line (*hν* = 1486.7 eV) was used from an Al anode. Scanning electron microscopy (SEM) was carried out on an EVO MA15 Zeiss system equipped with an energy dispersive X-ray detector (EDX). Thermogravimetric analysis (TGA) was carried out using a TA Q50 thermogravimetric analyzer with a heating rate of 10 °C min^–1^ in a nitrogen atmosphere. UL-94 vertical burning tests were carried out using a vertical burning instrument (Fire Testing Technology, UK). Specimen sheets for testing had dimensions of 130 × 13 × 0.6 mm^3^. Limiting oxygen index (LOI) tests were performed on samples with dimensions of 120 × 6.5 × 3 mm^3^ based on the standard test ASTM D 2863-77. The fire hazards of samples, with dimensions of 100 × 100 × 4 mm^3^, under forced-flaming conditions were detected using an FTT Cone Calorimeter according to ISO5660 under an external heat flux of 50 kW m^–2^. Dynamic mechanical analysis (DMA) was conducted on a Q800 DMA (TA Instruments, USA) from room temperature to 300 °C at a heating rate of 5 °C min^–1^ and at a frequency of 1 Hz in the tensile configuration. The tensile tests were carried out using an INSTRON 5966 dual column tabletop universal testing system according to the ASTM D3039-08 method, at a crosshead speed of 3.0 mm min^–1^.

## 3. Results and discussion

### 3.1 Characterization of pATH

**Scheme 1:**
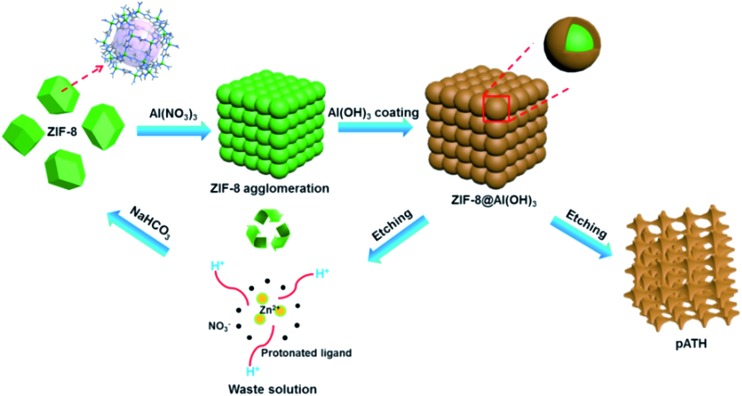
Schematic illustration of the synthetic procedure for pATH.

**Fig. 1:**
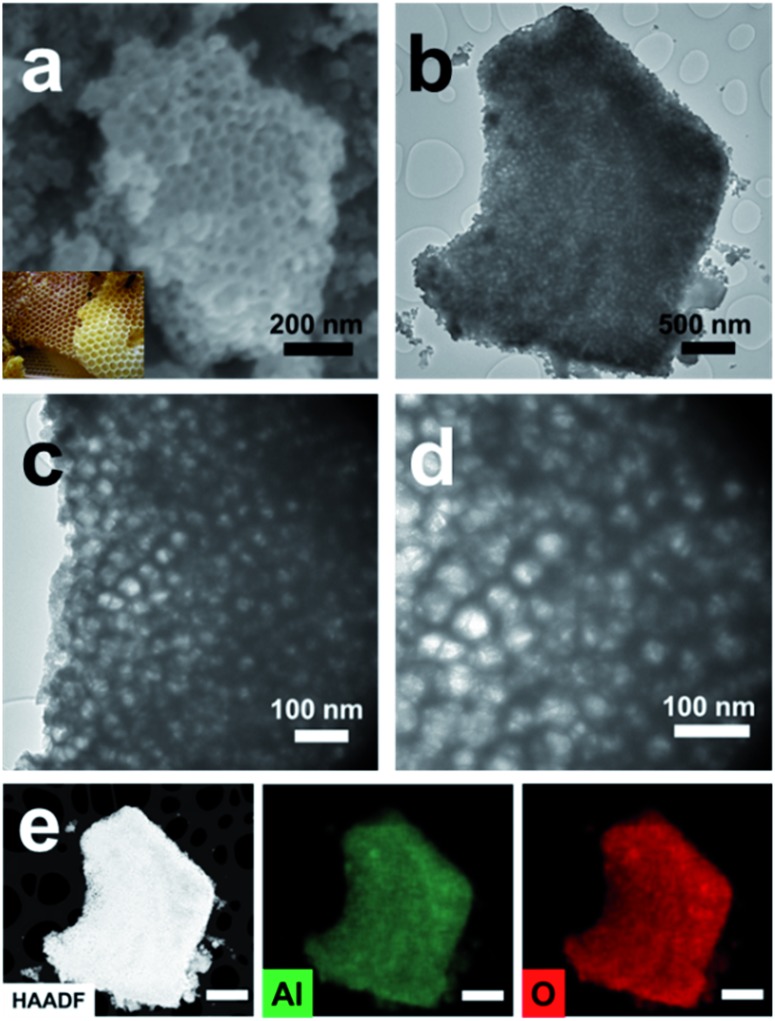
(a) SEM image (inset: digital photo of a honeycomb); (b) TEM image; (c and d) enlarged TEM images; (e) HAADF image and elemental mapping (scale bar: 500 nm) of pATH.

The preparation procedure of pATH is depicted in Scheme 1. The XRD pattern of the as-synthesized pATH (Fig. S1†) showed no characteristic peak, indicative of the amorphous feature for pATH which was in line with the previous report.^20^ The SEM image of commercial ATH is shown in Fig. S2.† The morphology of ATH corresponded to a large agglomeration of some smaller particles with no pores observed. The TEM image of the ZIF-8-2 h template is shown in Fig. S3,† which shows an octahedral shape with an average diameter of around 65 nm. The SEM image of pATH in Fig. 1a shows that pATH possessed a honeycomb-like porous structure, and the shape of each hole was faithfully inherited from the ZIF-8-2 h template. Elemental analysis of pATH by EDX, as shown in Fig. S4†, proved that aluminum and oxygen elements existed in the product. After further investigating the microstructure by TEM, the ordered porous structure of pATH, shown in Fig. 1b, was confirmed to be seemingly like a sieve. The enlarged TEM images in Fig. 1c and d showed that the individual pores were connected tightly one by one with a hexagonal wall. The mean pore size was circa 40 nm within the mesoporous range and the thickness of the wall was about 10 nm. The TEM images show some overlap of the mesopores in pATH, indicating that the pores resided in different layers. In the high angle annular dark field (HAADF) mode (Fig. 1e), a porous structure that existed in the thin edge of the particle was observed clearly by the sharp contrast. Elemental mapping showed that aluminum and oxygen elements were distributed uniformly in the particle. Furthermore, the EDX analysis from TEM, as shown in Fig. S5†, confirmed the results of SEM that pATH was composed of only aluminum and oxygen elements.

**Fig. 2:**
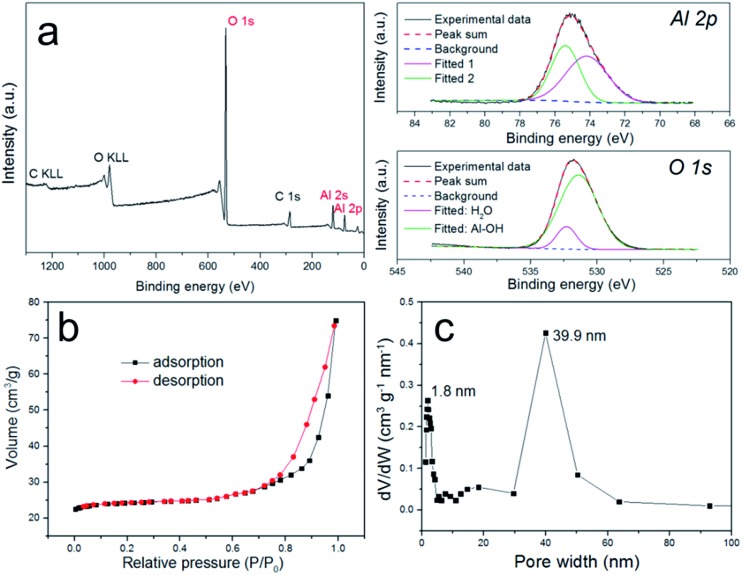
(a) XPS survey spectra and core-level scans of the Al and O elements of pATH; (b) N_2_ sorption isotherms of pATH; (c) pore size distribution of pATH.

The composition of pATH was also studied using XPS and FT-IR. In the XPS survey spectra of pATH shown in Fig. 2a, the elements in pATH were shown to include aluminum and oxygen without any impurities. The Al 2p peak in the core-level scan was separated into two symmetric single peaks. The presence of Al–OH in pATH was confirmed by the binding energy of the peak at 74.2 eV.^27^ The second transition peak at 75.3 eV might be associated with the amorphous phase of pATH.^28^ The peak of O 1s was also separated into two peaks. The fitted peak located at 531.4 eV for O 1s was ascribed to Al–O–H hydroxyl groups^27,29^ while the other smaller peak was caused by the adsorbed H_2_O in the system.^28^ FT-IR spectra of commercial ATH and pATH are shown in Fig. S6.† The as-synthesized pATH was extremely pure with an intensive band at around 3470 cm^–1^ which was assigned to the Al–O–H stretching vibration,^30^ a peak near 1038 cm^–1^ which was assigned to the asymmetric stretching of the internal AlO_4_ tetrahedral entity, and a broad band at 608 cm^–1^ which was indexed as the symmetric bending of the Al–O bond.^21^

The N_2_ adsorption–desorption isotherms shown in Fig. 2b provide detailed information about the porous structure of pATH. The well-defined H3-type hysteresis loop at high relative pressures was indicative of a typical type IV isotherm curve, which is characteristic for mesoporous materials. The H3 hysteresis loop is traditionally associated with slit-shaped pores,^31^ but it was also recently detected in hollow particles with porous walls.^32^ An H3 hysteresis loop might appear in the case of hierarchical porous materials with multimodal nature pore size distributions, and the formation of a hysteresis loop is mainly a result of the relatively large pores.^33,34^ Similarly, in this study the pore widths from the pore size distribution were estimated, using a non-local density functional theory model, to be centered at both 1.8 nm and 39.9 nm. The size of the mesopores (39.9 nm) in pATH was consistent with the TEM results. According to the report by Sivaniah *et al.*, the pore size of ZIF-8 was around 1–2 nm.^35^ Therefore, the formation of homogeneous micropores (1.8 nm) in the product was probably due to uniform replication of the intrinsic pores of the ZIF-8 template on the aluminum hydroxide walls, which is reminiscent of a previous report that found that the micropores of microporous titania were derived from the MOF template.^36^ The computed BET surface area of pATH was 37.1 m^2^ g^–1^, which is more than 10 times that of solid commercial ATH (3.2 m^2^ g^–1^). Because of the abundant porosity of pATH, its density was lower than that of the pyknotic nonporous ATH, which is demonstrated by the digital photos shown in Fig. S7.† The light pATH tended to uniformly disperse in the polymer matrix. Meanwhile, the weight loss of pATH at 780 °C, shown in the TGA curve in Fig. S8†, was revealed to be around 35% due to the escape of water contained in Al(OH)_3_, thus conforming to a stoichiometric ratio similar to that of commercial ATH. All-sided characterization corroborated that the as-synthesized product, pATH, was a honeycomb-like mesoporous aluminum hydroxide with a relatively high specific surface area. The mesopores of pATH were also altered into macropores by tuning the size of the ZIF-8 template. Fig. S9† shows electron microscopy images of the macro-pATH obtained by using the ZIF-8-5 h template. Along with the increase in template size, the pore width of the product expanded as well. The mean pore size of macro-pATH was around 70 nm, which is within the macroporous range. These results suggest that the pore sizes of pATH were easily modulated by changing the dimensions of the ZIF-8 template. Except for the difference in pore size, macro-pATH adopted a similar structure and the same elemental composition to that of pATH.

**Fig. 3:**
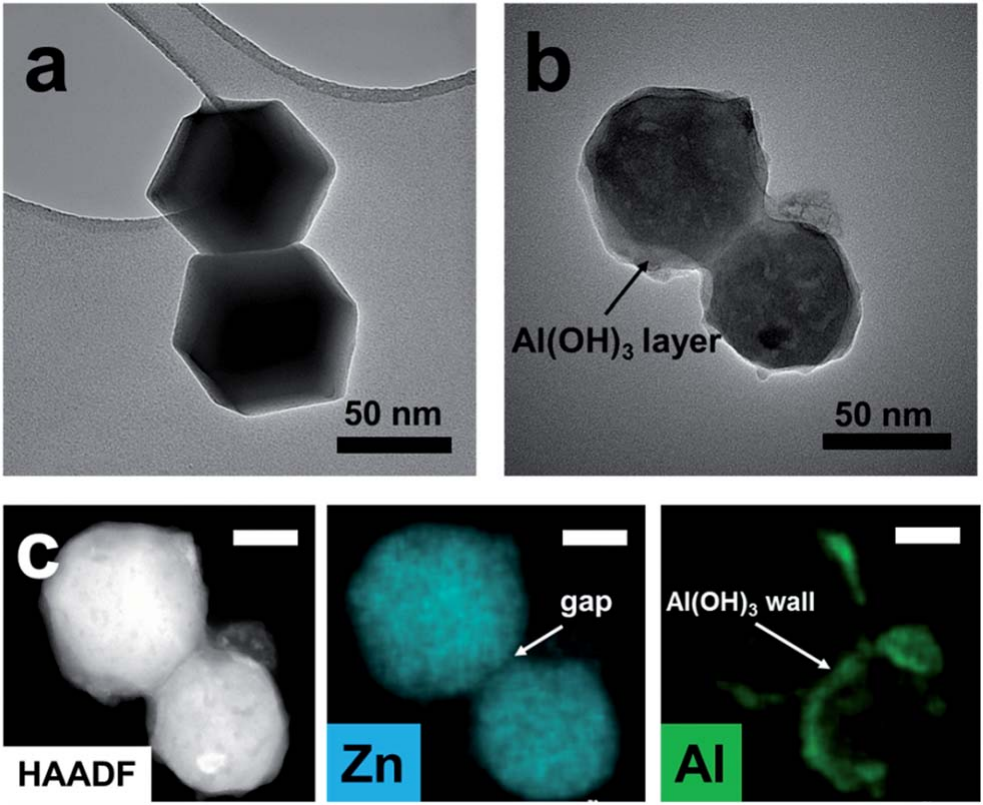
(a) TEM image of the ZIF-8-2 h template; (b) TEM image of the intermediate gathered after a reaction time of 10 min; (c) HAADF image and elemental mapping (scale bar: 20 nm).

In order to investigate the formation mechanism of pATH, the intermediate was gathered after the reaction time reached 10 min, and to make it simple and clear, we selected a dimer instead of a large agglomeration. The dimer of the pure template had a regular hexagonal shape, as shown in Fig. 3a, however, when the reaction time reached 10 min, the boundary of the dimer became ambiguous owing to decomposition of the template. Moreover, the existence of multiple holes in the particle compared with the previous solid structure also confirmed this resolution. From the elemental mapping analysis, it could be observed that zinc element was coated by aluminum element, and a gap between the two particles in the dimer appeared, filling with aluminum hydroxide to become the pore walls. As we know, aluminum nitrate hydrolyzes slowly in water according to the equation below:Al^3+^ + 3H_2_O ⇌ Al(OH)_3_↓ + 3H^+^

The ethanol–water solution rendered the hydrolysis gradual, with a slow rate and a uniform layer of amorphous aluminum hydroxide generated on the ZIF-8 template. The interiors of ZIF-8 nanocrystals are known to be strongly hydrophobic, however, their exterior surfaces are hydrophilic due to the existence of terminal N–H functional groups.^37^ We postulated that this provides an abundance of H-bonding and coordination sites that stimulate the coating of aluminum hydroxide on the ZIF-8 surface, which is similar to the report that aluminum hydroxide coated on graphene and cloned the morphology of the nanosheet.^20,38^ With the hydrolysis proceeding, a large amount of H^+^ was produced. The high H^+^ concentration gradient gave rise to severe agglomeration of the ZIF-8 particles which generated a number of monoliths. In addition, accompanied by the generation of an aluminum hydroxide shell, the protonation of the ligand resulted in the simultaneous decomposition of the ZIF-8 core. The inward stress strain made the aluminum hydroxide framework contract slightly. Since the formation of aluminum hydroxide and decomposition of ZIF-8 occurred at the same time, with the disappearance of ZIF-8 in the interfacial region adjacent to the aluminum hydroxide shell, newly generated aluminum hydroxide was supplied to fill the interface until termination of the hydrolysis reaction. Thus, different from traditional template methods, the final pore size of the product prepared *via* our “amorphous replica method” was smaller than the dimensions of the ZIF-8 template. Furthermore, because the H^+^ ions were consumed continuously by the protonation process, the hydrolysis reaction proceeded until total decomposition of the ZIF-8 template. As we know, the hydrolysis of aluminum nitrate is extremely slow, while in this case the continuous consumption of H^+^ ions enabled the hydrolysis reaction to proceed rapidly. Finally, the nanoporous aluminum hydroxide framework was produced, with the pores duplicating the morphology of the polyhedra based on the proposed “amorphous replica method”, and the ZIF-8 template was removed synchronously without leaving any residue. As simple as the synthesis procedure seemed, it is interesting that no researchers before had noticed the probability of the formation of mesoporous aluminum hydroxide from an ordinary hydrolysis reaction.^39^ We considered that this might be due to two main reasons: firstly, the hydrolysis of aluminum nitrate is considerably slow, and therefore this precursor is presumed to be a useless candidate for the preparation of aluminum hydroxide *via* hydrolysis, and secondly, the choice of a self-decomposing ZIF template under acidic conditions is fairly crucial. In view of our results, ZIF-8 was found to be a suitable template that decomposed completely while ZIF-67, a cobalt-based MOF, was more stable than ZIF-8 under acidic conditions and, due to the hydrolysis of metal nitrates, layered double hydroxide nanocages were ultimately produced instead.^40^

**Fig. 4:**
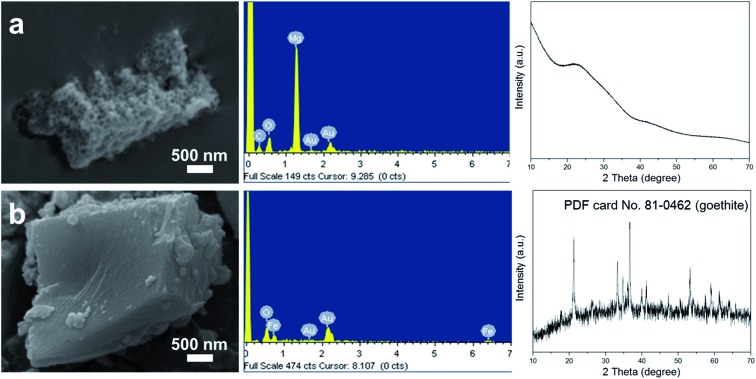
SEM images, EDX spectra and XRD patterns of magnesium hydroxide (a) and iron hydroxide (b).

In order to further demonstrate the above hypothesis and study the general applicability of this method, iron nitrate and magnesium nitrate were selected to replace aluminum nitrate to produce their corresponding hydroxides. As shown in Fig. 4a, the sample synthesized when using magnesium nitrate as the precursor also exhibited a porous structure, as revealed by SEM, and the components of the sample were Mg and O, as found from analysis of the EDX spectrum. Interestingly, the XRD pattern of this product also presented an amorphous phase, which resembled that of pATH. After calcination at a high temperature, sharp peaks appeared in the XRD pattern of the sample, as shown in Fig. S10a†, which were indexed to magnesium oxide (MgO) in the brucite phase (JCPDS card No. 02-1395). As a result, the product prepared when using magnesium nitrate as the precursor was found to be amorphous nanoporous magnesium hydroxide [Mg(OH)_2_]. Meanwhile, calcination of pATH was also carried out and peaks assigned to α-alumina (JCPDS card No. 50-0741) emerged in the XRD pattern, as shown in Fig. S10b.† Meanwhile, the porous structures of the two hydroxides remained after calcination, as shown in the SEM images, which indicates that it is possible to form nanoporous magnesium oxide and alumina by means of this method. However, when iron nitrate was used as the precursor, a compact solid monolith without pores was detected, as demonstrated in the SEM image shown in Fig. 4b. The XRD pattern showed that the crystalline phase of the product was iron oxide (FeOOH) in the goethite phase (JCPDS card No. 81-0462). Obviously, it was difficult to produce amorphous hydroxide using this metal salt precursor under the current conditions. The rapid formation of large crystals could not entirely cover the surface of the ZIF-8 polyhedra template. These results illustrated that only amorphous hydroxide afforded the fascinating nanoporous microstructure, which is in line with the previous report.^20^

**Fig. 5:**
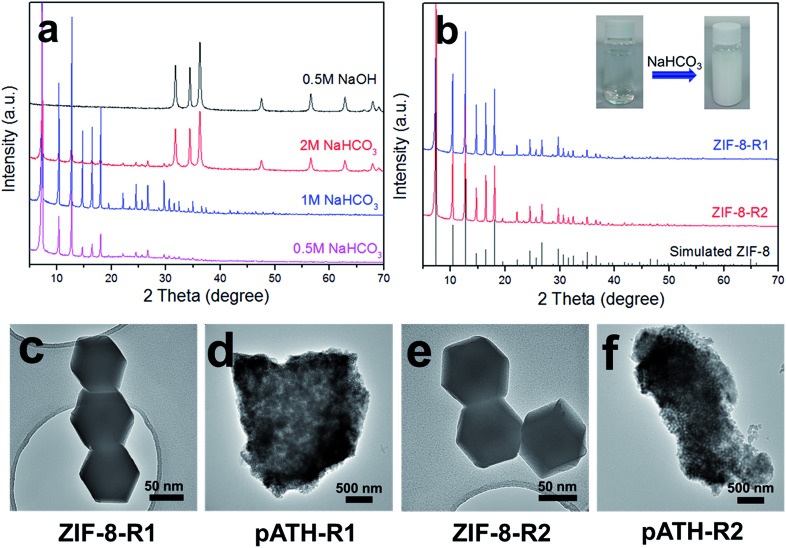
(a) XRD patterns of the recycled products by different alkalis; (b) XRD patterns of recycled ZIF-8 by NaHCO_3_; (c–f) TEM images of recycled ZIF-8 and pATH synthesized using the recycled templates.

Another attractive aspect of our work was the recycling of the ZIF-8 template from the waste solution by alkali. The imidazole may be deprotonated under alkaline conditions and re-chelate with the Zn ion again. Herein, three different kinds of alkalis were chosen for the recovery of ZIF-8: NaOH, NaHCO_3_ and NH_3_·H_2_O. After adding 0.5 M NaOH, a white precipitate appeared immediately in the solution. The XRD pattern in Fig. 5a shows that the precipitate was hexagonal zinc hydroxide [Zn(OH)_2_, JCPDS card No. 48-1066]. The failure of the recycling was caused by the strong alkaline nature of NaOH, whose OH^–^ associated with the zinc ion directly instead of with the protonated imidazole, and therefore Zn(OH)_2_ was the only product. When ammonia solution was used, the waste solution remained clear and nothing happened because the zinc ions chelated with amino groups to form soluble [Zn(NH_3_)^4^]^2+^ instead of with the imidazole ligand. Things changed for the better when NaHCO_3_, a mild alkali, was used to recycle ZIF-8. The XRD pattern, shown in Fig. 5a, of the product recycled by 0.5 M NaHCO_3_ was ascribed to that of ZIF-8. Upon increasing the NaHCO_3_ concentration to 1 M, the XRD pattern remained the same with enhanced intensity peaks. However, a Zn(OH)_2_ phase arose, caused by an excess of OH^–^ ions, when the concentration was increased to 2 M, the peaks of which mixed with the ZIF-8 peaks in the XRD pattern. Therefore, 1 M NaHCO_3_ was the optimized experimental concentration for the recycling of ZIF-8 from the waste solution after the preparation of pATH. Under these conditions, ZIF-8 was recycled (ZIF-8-R1) and used to prepare pATH (pATH-R1) again. Similarly, the procedure was repeated once more and the products (ZIF-8-R2 and pATH-R2) were obtained again. The XRD patterns of ZIF-8-R1 and ZIF-8-R2 shown in Fig. 5b were identical to that of the simulated ZIF-8. The inset images show the color change after addition of NaHCO_3_ to the waste solution as an example. The TEM images in Fig. 5c–f demonstrated that the recycled ZIF-8 possessed similar morphology and dimensions to those of the original one, and there were no obvious distinctions between pATH-R1 (R2) and pATH. According to these results, we can conclude that the preparation of pATH was a sustainable and cost-effective route due to the recyclability of the template, which is of benefit for practical applications.

As a proof-of-concept application of pATH, DOPO encapsulated pATH, with the help of a silane coupling agent and modified pATH, was exploited as a flame retardant for epoxy resin. In the FT-IR spectra of KH–DOPO, ATH–DOPO and pATH–DOPO in Fig. S6,† it can be clearly seen that after the modification of KH–DOPO, the peaks at around 2900 cm^–1^, attributed to C–H, and 1594 cm^–1^, assigned to biphenyl in KH–DOPO, were also found in the ATH–DOPO and pATH–DOPO spectra.^26^ The TGA profiles in Fig. S8† show that the modified ATH and pATH had higher decomposition temperatures than those of the unmodified ones because DOPO decomposed at a temperature of between 300 °C and 400 °C, which demonstrated that it is more thermally stable than aluminum hydroxide. Fig. S11† shows the SEM images and EDX spectra of ATH–DOPO and pATH–DOPO. In the SEM image of ATH–DOPO, the product was shown to be made up by agglomerated small particles, with no big difference observed in comparison with the unmodified one. With respect to pATH–DOPO, it was still possible to detect the porous structure of the material even after modification by DOPO. DOPO possibly entered into the pore channel of pATH, which meant that pATH loaded more phosphorous flame retardant in comparison with the solid commercial ATH. The EDX spectra confirmed this hypothesis on the basis of the Al to P atomic ratio, and the results were almost the same when choosing different check points on the particle. pATH showed a higher phosphorous loading content than that of ATH, thus disclosing its potential as a high-efficiency flame retardant in epoxy resin. The peaks of silicon element in the two samples corresponded to the silicon contained in the silane coupling agent.

### 3.2 Flame retardancy of epoxy composites

**Fig. 6:**
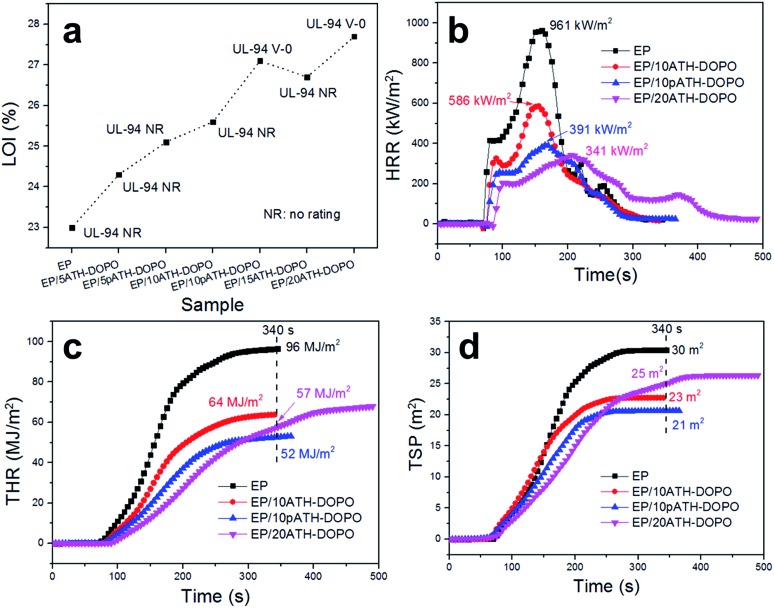
(a) LOI values and UL-94 results for epoxy composites; (b) HRR curves of epoxy composites; (c) THR curves of epoxy composites; (d) TSP curves of epoxy composites.

The limiting oxygen index (LOI) is the minimum oxygen content to sustain the burning of materials.^41^ The LOI values of the neat epoxy resin and its composites containing different amounts of ATH–DOPO and pATH–DOPO are shown in Fig. 6a. In order to simplify, EP/5ATH–DOPO corresponds to epoxy resin with 5 wt% ATH–DOPO and others were named similarly. The LOI value of neat epoxy resin was 20.0% and upon increasing the loading amount of filler the value kept rising. For the EP/10pATH–DOPO sample, the LOI value was 27.1% higher than that of the sample containing 10% ATH–DOPO (25.6%), which is close to the value of EP/20ATH–DOPO (27.7%). UL-94 tests are commonly used to evaluate the flammability of materials. All the samples presented no rating in the tests except for EP/10pATH–DOPO and EP/20ATH–DOPO. These two samples passed the V-0 rating for the UL-94 tests, thus illustrating their relatively low flammability. Combining the LOI and UL-94 results, 10pATH–DOPO showed a similar efficiency as a flame retarding epoxy resin to that of 20ATH–DOPO. Therefore, cone calorimeter tests were carried out on epoxy containing these two flame retardants to monitor the fire behaviours of the composites. EP/10ATH–DOPO was also taken into consideration as a contrast.

The heat release rate (HRR), total heat release (THR) and total smoke production (TSP) curves are depicted in Fig. 6b–d, respectively. The neat epoxy resin burned fiercely once ignited and its HRR curve exhibited a sharp peak after 100 s of combustion time. The peak of HRR (PHRR) for the neat epoxy resin without any flame retardant reached 961 kW m^–2^. In the presence of 10ATH–DOPO, the PHRR of the composite decreased to 586 kW m^–2^, and, with an increased addition amount, EP/20ATH–DOPO showed a PHRR value of 341 kW m^–2^. The addition of 10pATH–DOPO reduced the PHRR value of the composite strikingly to 391 kW m^–2^, which is close to the result of EP/20ATH–DOPO. Meanwhile, the times to ignition (TTI) of these three samples were all longer compared with that of the neat one. Although EP/20ATH–DOPO had the lowest PHRR, a second broad peak appeared in the HRR curve, probably because the formed char layer was not robust enough to prevent the escape of combustible volatiles, thus leading to further burning of the underlying polymer. This consequence was reflected exactly in the THR curves in Fig. 6c. The THR value of EP/20ATH–DOPO increased continuously and at 340 s the value was 57 MJ m^–2^, which is higher than that of the 10pATH–DOPO blended sample. 10pATH–DOPO effectively cut down the THR value from 96 MJ m^–2^ in the neat epoxy resin to 52 MJ m^–2^ at 340 s, and presented the lowest THR value among all the samples at that time. TSP is also an important parameter for estimating the fire hazards of polymeric materials, because smoke causes deaths in fire accidents. 10ATH–DOPO reduced the smoke emission of the composite efficiently from 30 m^2^ to 23 m^2^ in comparison with the neat epoxy. However, although the addition of 20ATH–DOPO slowed down the smoke production rate of the composite in terms of the low slope of the TSP curve, the total smoke production of the composite was higher than that of the sample with a lower loading amount of ATH–DOPO. The EP with 10pATH–DOPO possessed the lowest THR value of 21 m^2^ at 340 s among all the samples, which is indicative of relatively low smoke hazards. The results of EP/10pATH–DOPO were able to rival those of EP/20ATH–DOPO because of the high specific surface area, mesoporous structure and low density of pATH which are beneficial for better interaction with the epoxy matrix and a higher loading amount of phosphorous flame retardant.

### 3.3 Mechanical properties of epoxy composites

**Fig. 7:**
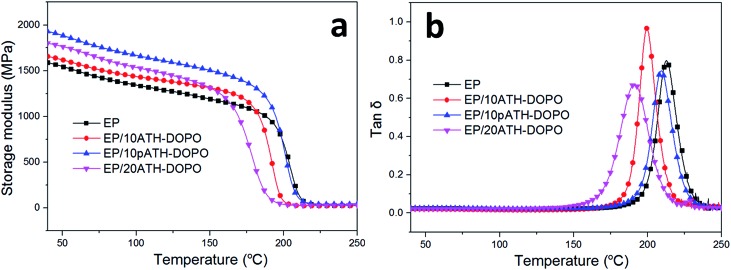
Dynamic mechanical curves for epoxy and its composites: (a) storage modulus; (b) tan *δ*.

The dynamic mechanical behaviours of the epoxy composites were studied by DMA and the results are shown in Fig. 7. The storage modulus of the composite primarily depended on the stiffness of the additives and the interactions between the fillers and polymer matrix. The addition of the flame retardants increased the storage modulus of the composites in comparison with those of the neat ones due to the greater stiffness of the inorganic ATH relative to the soft epoxy resin. The storage modulus was the highest for EP/10pATH–DOPO out of all the samples in the glass state. This was partly because of the good dispersibility of pATH–DOPO which interacts well in the matrix and because it is endowed with a high specific surface area, abundant porosity and low density of pATH.^42^ The glass transition temperature (*T*_g_) was obtained from the peak value of the tan *δ* curve. As shown in Fig. 7b, the incorporation of DOPO modified ATH and pATH caused a decrease in *T*_g_ of the composite, probably because DOPO reduced the cross-linking density of the epoxy resin by elevating the activation energy of the curing reaction between the curing agent and the polymer matrix.^43^ The obvious decrease in *T*_g_ for the ATH–DOPO blended composites was attributed to the large agglomeration of DOPO modified ATH particles, rendering a decrease in the amount of filler in the epoxy with an enhanced confinement effect between the particles.^42^ In contrast, a slight decrease in *T*_g_ was observed for the pATH–DOPO mixed sample, indicating good dispersion of the filler with more particles in the unit area of the polymer matrix.

**Fig. 8:**
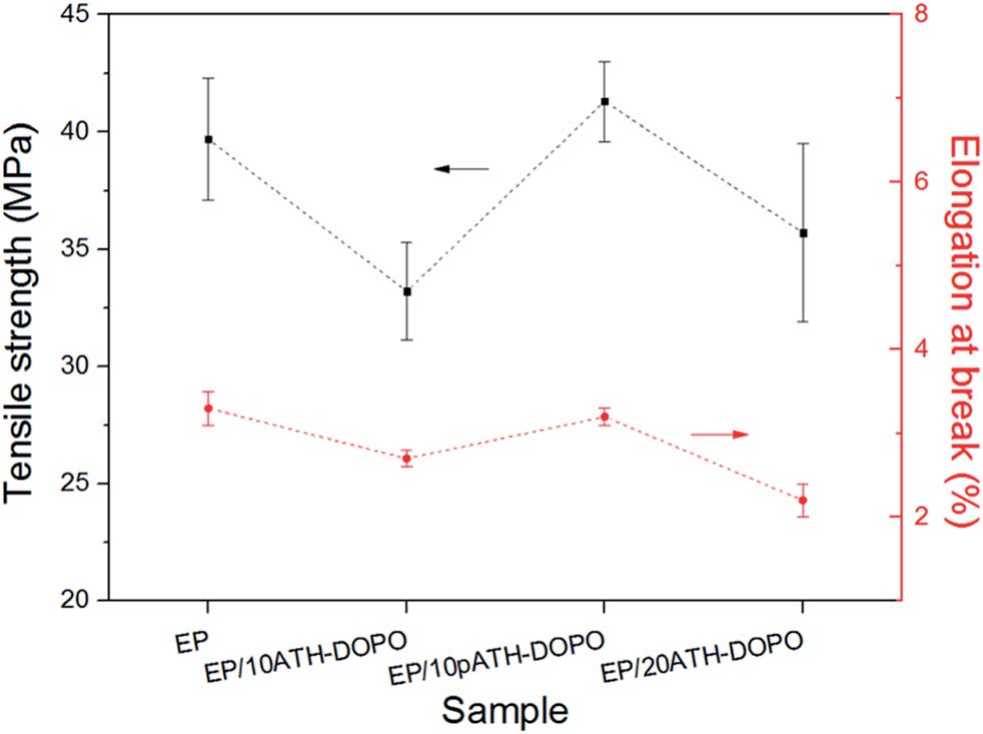
Tensile strength and elongation at break for epoxy and its composites.

To further compare the influence of the filler on the mechanical properties of the epoxy composites, tensile tests were carried out. The tensile strength and elongation at break results are presented in Fig. 8. The tensile strength for neat epoxy was 39.7 MPa, and the elongation at break was 3.3%. Upon the addition of 10ATH–DOPO and 20ATH–DOPO, the tensile strengths of the composites decreased to 33.2 MPa and 35.7 MPa, respectively. In contrast, the tensile strength of EP/10pATH–DOPO increased slightly to 41.3 MPa. Meanwhile, the elongation at break of the composite containing pATH–DOPO only decreased a little to 3.2%, in contrast to the rather obvious decreases for EP/10ATH–DOPO and EP/20ATH–DOPO. The relatively high loading amount of filler in EP/20ATH–DOPO ruined the tensile properties of the composite, while the possibly formed agglomeration of ATH–DOPO, due to the low BET surface area, conferred poor dispersion in the polymer matrix to 10ATH–DOPO, thus resulting in the weak tensile performance of the composite. Moreover, 10pATH–DOPO presented better results because of its uniform distribution and good interaction with the matrix in the epoxy owing to the low density, abundant porosity and high specific surface area of pATH.^44,45^

## 4. Conclusions

To conclude, an acid-sensitive self-decomposing MOF, ZIF-8, was subtly designated as an easily removable template for the preparation of honeycomb-like mesoporous aluminum hydroxide by a one step reaction, according to the proposed “amorphous replica method”, for the first time. The template was removed synchronously, without any tedious or dangerous processes, as soon as the product was generated. Furthermore, the pore sizes of the product were facilely modulated by tuning the dimensions of the ZIF-8 polyhedra. It was worth noting that the ZIF-8 template was recoverable from the waste solution after synthesis *via* deprotonation of the ligand by a mild alkali. The recycled ZIF-8 was reused to prepare pATH again, thus forming a delicate synthesis cycle. The renewable template and facile preparation method presented in this work cater to the demand for nanoporous hydroxides for practical applications. The flame retardancy of EP/10pATH–DOPO rivals that of EP/20ATH–DOPO, and the former presented better mechanical properties. Since nanoporous materials have been widely applied in photoelectric devices,^46^ medicinal chemistry,^47^ adsorbents^48^ and so forth, the potential applications in other areas of the thus-obtained unique material will be further investigated in our future work.

## References

[cit1] Jia C.-J., Schwickardi M., Weidenthaler C., Schmidt W., Korhonen S., Weckhuysen B. M., Schüth F. (2011). J. Am. Chem. Soc..

[cit2] Niu W., Li L., Liu X., Wang N., Liu J., Zhou W., Tang Z., Chen S. (2015). J. Am. Chem. Soc..

[cit3] Lin L., Xu X., Chu C., Majeed M. K., Yang J. (2016). Angew. Chem., Int. Ed..

[cit4] Song Q., Jiang S., Hasell T., Liu M., Sun S., Cheetham A. K., Sivaniah E., Cooper A. I. (2016). Adv. Mater..

[cit5] Yang D., Lu Z., Rui X., Huang X., Li H., Zhu J., Zhang W., Lam Y. M., Hng H. H., Zhang H. (2014). Angew. Chem., Int. Ed..

[cit6] Jiao F., Jumas J.-C., Womes M., Chadwick A. V., Harrison A., Bruce P. G. (2006). J. Am. Chem. Soc..

[cit7] Wang C. W., Yang S., Fang W. Q., Liu P., Zhao H., Yang H. G. (2015). Nano Lett..

[cit8] Liu S., Wang F., Dong R., Zhang T., Zhang J., Zhuang X., Mai Y., Feng X. (2016). Adv. Mater..

[cit9] Luo Z., Miao R., Huan T. D., Mosa I. M., Poyraz A. S., Zhong W., Cloud J. E., Kriz D. A., Thanneeru S., He J., Zhang Y., Ramprasad R., Suib S. L. (2016). Adv. Energy Mater..

[cit10] Morozan A., Jaouen F. (2012). Energy Environ. Sci..

[cit11] Zhang W., Wu Z.-Y., Jiang H.-L., Yu S.-H. (2014). J. Am. Chem. Soc..

[cit12] Shang L., Yu H., Huang X., Bian T., Shi R., Zhao Y., Waterhouse G. I. N., Wu L. Z., Tung C. H., Zhang T. (2016). Adv. Mater..

[cit13] You S., Gong X., Wang W., Qi D., Wang X., Chen X., Ren N. (2016). Adv. Energy Mater..

[cit14] Salunkhe R. R., Tang J., Kobayashi N., Kim J., Ide Y., Tominaka S., Kim J. H., Yamauchi Y. (2016). Chem. Sci..

[cit15] Yu L., Yang J. F., Lou X. W. D. (2016). Angew. Chem., Int. Ed..

[cit16] Hu H., Zhang J., Guan B., Lou X. W. D. (2016). Angew. Chem., Int. Ed..

[cit17] Wang X., Zhao S., Zhang Y., Wang Z., Feng J., Song S., Zhang H. (2016). Chem. Sci..

[cit18] Sun C.-Y., Qin C., Wang X.-L., Yang G.-S., Shao K.-Z., Lan Y.-Q., Su Z.-M., Huang P., Wang C.-G., Wang E.-B. (2012). Dalton Trans..

[cit19] Zheng H., Zhang Y., Liu L., Wan W., Guo P., Nyström A. M., Zou X. (2016). J. Am. Chem. Soc..

[cit20] Huang Z., Zhou A., Wu J., Chen Y., Lan X., Bai H., Li L. (2016). Adv. Mater..

[cit21] Liu Y., He J., Yang R. (2015). Ind. Eng. Chem. Res..

[cit22] Qin Z., Li D., Li Q., Yang R. (2016). Mater. Des..

[cit23] Haile M., Fomete S., Lopez I. D., Grunlan J. C. (2016). J. Mater. Sci..

[cit24] Wang X., Kalali E. N., Wang D.-Y. (2016). Nano Adv..

[cit25] Wei J., Liang Y., Hu Y., Kong B., Zhang J., Gu Q., Tong Y., Wang X., Jiang S. P., Wang H. (2016). Angew. Chem., Int. Ed..

[cit26] Wang Q., Xiong L., Liang H., Chen L., Huang S. (2016). Polym. Compos..

[cit27] Iatsunskyi I., Kempiński M., Jancelewicz M., Załęski K., Jurga S., Smyntyna V. (2015). Vacuum.

[cit28] Kloprogge J. T., Duong L. V., Wood B. J., Frost R. L. (2006). J. Colloid Interface Sci..

[cit29] Lee J. S., Kim H. S., Park N.-K., Lee T. J., Kang M. (2013). Chem. Eng. J..

[cit30] Pan L., Ji Q., Qin Y., Jiang Y., Zhang Z., Zhang S., Wang Z. (2013). RSC Adv..

[cit31] Xiong Z., Liao C., Wang X. (2014). J. Mater. Chem. A.

[cit32] Jia R., Chen J., Zhao J., Zheng J., Song C., Li L., Zhu Z. (2010). J. Mater. Chem. A.

[cit33] Lou X. W., Deng D., Lee J. Y., Archer L. A. (2008). J. Mater. Chem. A.

[cit34] Guo S., Li D., Zhang L., Li J., Wang E. (2009). Biomaterials.

[cit35] Song Q., Nataraj S. K., Roussenova M. V., Tan J. C., Hughes D. J., Li W., Bourgoin P., Alam M. A., Cheetham A. K., Al-Muhtaseb S. A., Sivaniah E. (2012). Energy Environ. Sci..

[cit36] Hall A. S., Kondo A., Maeda K., Mallouk T. E. (2013). J. Am. Chem. Soc..

[cit37] Zhang K., Lively R. P., Zhang C., Koros W. J., Chance R. R. (2013). J. Phys. Chem. C.

[cit38] Yang H., Bradley S. J., Chan A., Waterhouse G. I. N., Nann T., Kruger P. E., Telfer S. G. (2016). J. Am. Chem. Soc..

[cit39] Yang L.-P., Lin X.-J., Zhang X., Zhang W., Cao A.-M., Wan L.-J. (2016). J. Am. Chem. Soc..

[cit40] Jiang Z., Li Z., Qin Z., Sun H., Jiao X., Chen D. (2013). Nanoscale.

[cit41] Li C., Wan J., Kalali E. N., Fan H., Wang D.-Y. (2015). J. Mater. Chem. A.

[cit42] Jiajun M., Junxiao Y., Yawen H., Ke C. (2012). J. Mater. Chem. A.

[cit43] Liu S., Chen J., Zhao J., Jiang Z., Yuan Y. (2015). Polym. Int..

[cit44] Pan Y.-T., Trempont C., Wang D.-Y. (2016). Chem. Eng. J..

[cit45] Jonoobi M., Aitomäki Y., Mathew A. P., Oksman K. (2014). Composites, Part A.

[cit46] Chen B., Susha A. S., Reckmeier C. J., Kershaw S. V., Wang Y., Zou B., Zhong H., Rogach A. L. (2017). Adv. Mater..

[cit47] Cortez A., Li Y., Miller A. T., Zhang X., Yue K., Maginnis J., Hampton J., Hall D. S., Shapiro M., Nayak B. (2016). J. Med. Chem..

[cit48] Yan Y., Li W., Yang J., Zheng A., Liu F., Feng X., Sparks D. L. (2014). Environ. Sci. Technol..

