# Supplementary material for: Interfacial engineering of renewable metal organic framework derived honeycomb-like nanoporous aluminum hydroxide with tunable porosity
Source: Chem Sci. 2017 Feb 28;8(5):3399–409. doi: 10.1039/c6sc05695d (PMC5417008; doi:10.1039/c6sc05695d)
Supplement: Supplementary file 1 [file SC-008-C6SC05695D-s001.pdf]

Electronic Supplementary Material (ESI) for Chemical Science.
This journal is © The Royal Society of Chemistry 2017

Supplementary Information for

# Interfacial engineering of renewable metal organic framework derived honeycomb-like nanoporous aluminum hydroxide with tunable porosity and its application

Ye-Tang Pan, Lu Zhang, Xiaomin Zhao, De-Yi Wang*

IMDEA Materials Institute, C/Eric Kandel, 2, 28906 Getafe, Madrid, Spain
Corresponding Author: E-mail address: deyi.wang@imdea.org

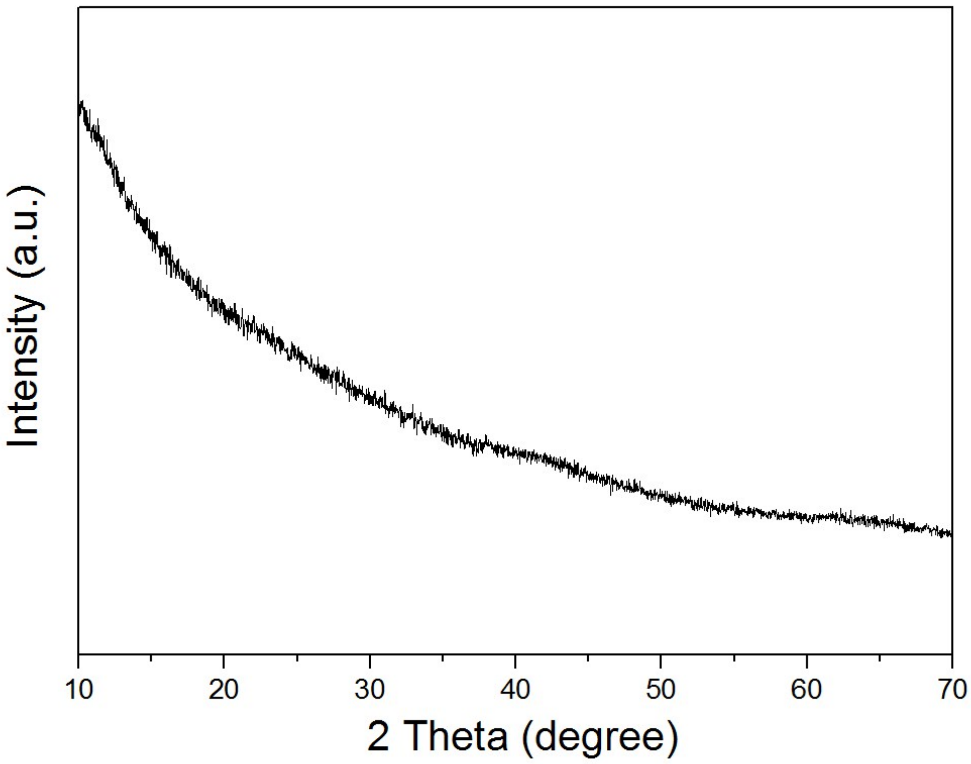


**Fig. S1** XRD pattern of pATH.

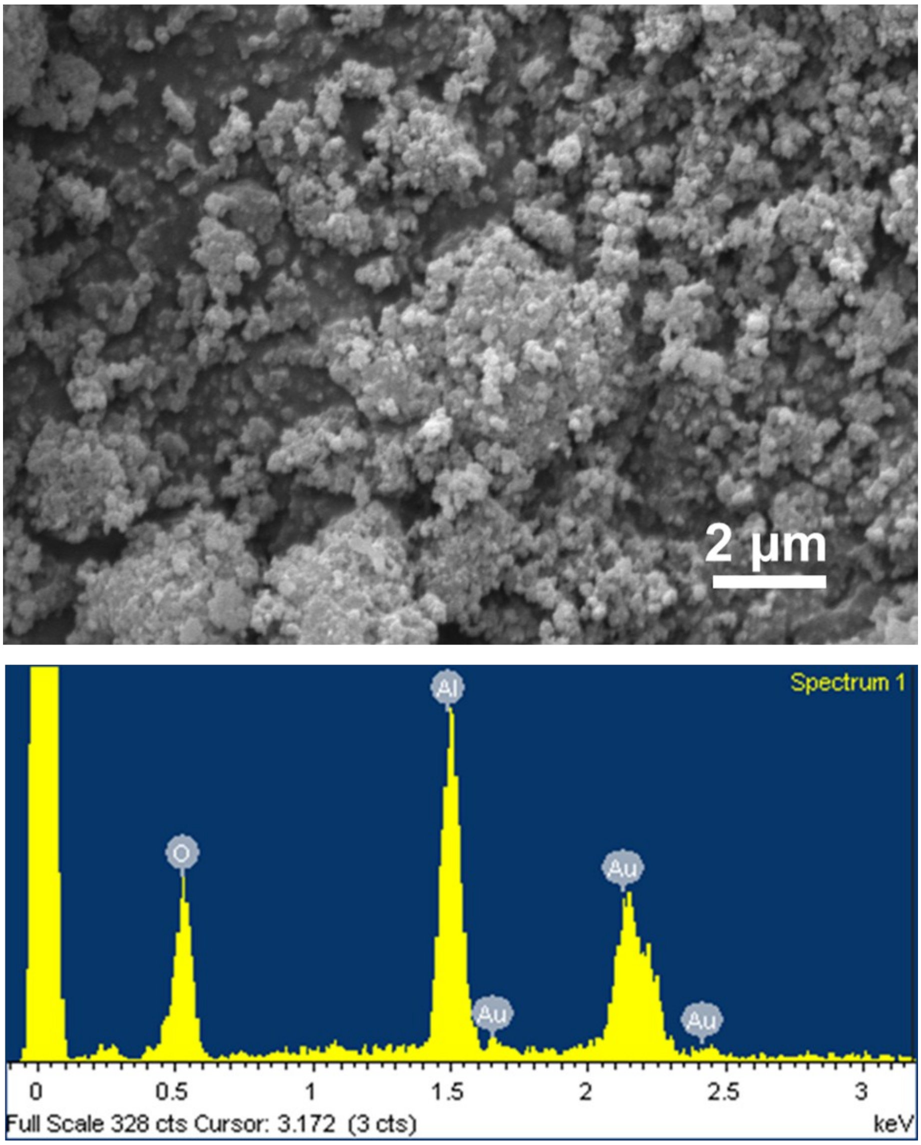


**Fig. S2** SEM image and EDX spectra of commercial ATH.

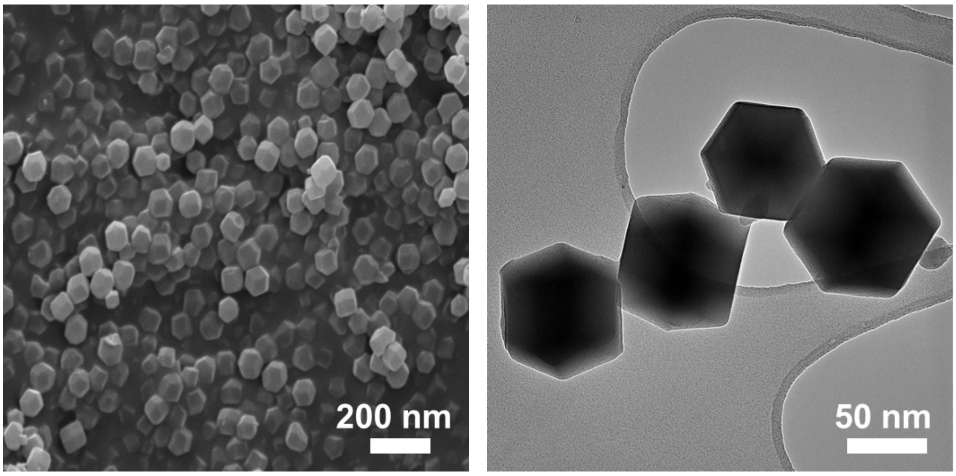


**Fig. S3** SEM and TEM images of ZIF-8-2h tempalte.

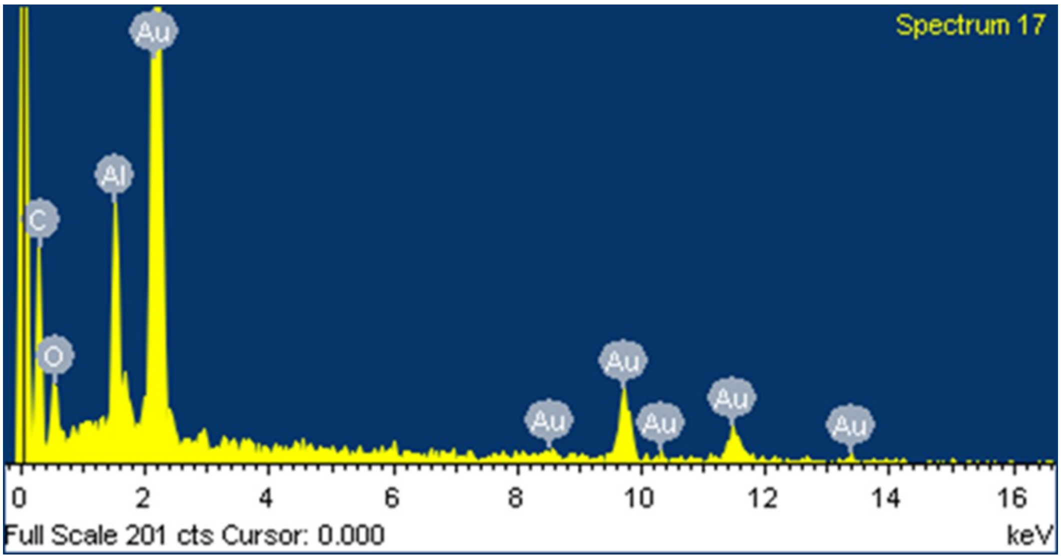


**Fig. S4** EDX spectra of pATH from SEM. (C element was derived from the conductive belt)

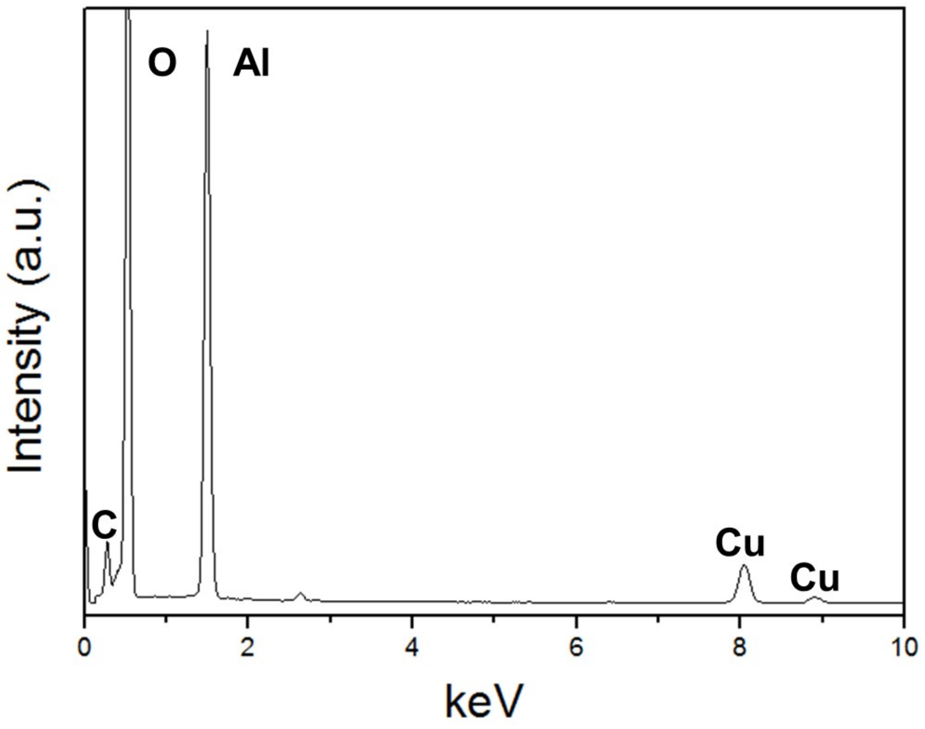


**Fig. S5** EDX spectra of pATH from TEM.

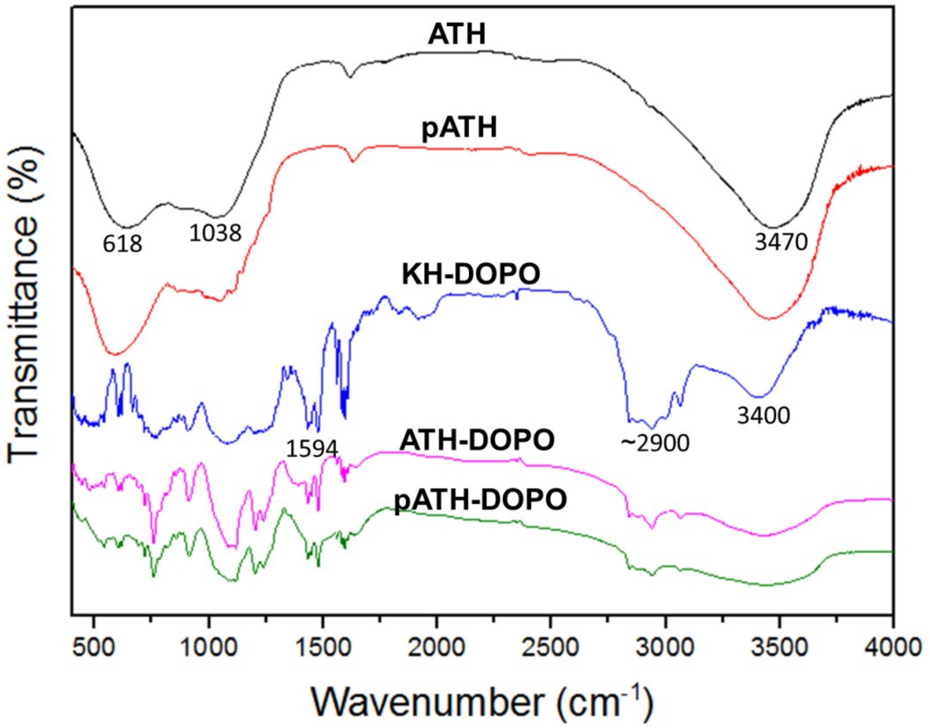


**Fig. S6** The FT-IR spectra of ATH, pATH, KH-DOPO, ATH-DOPO and pATH-DOPO.

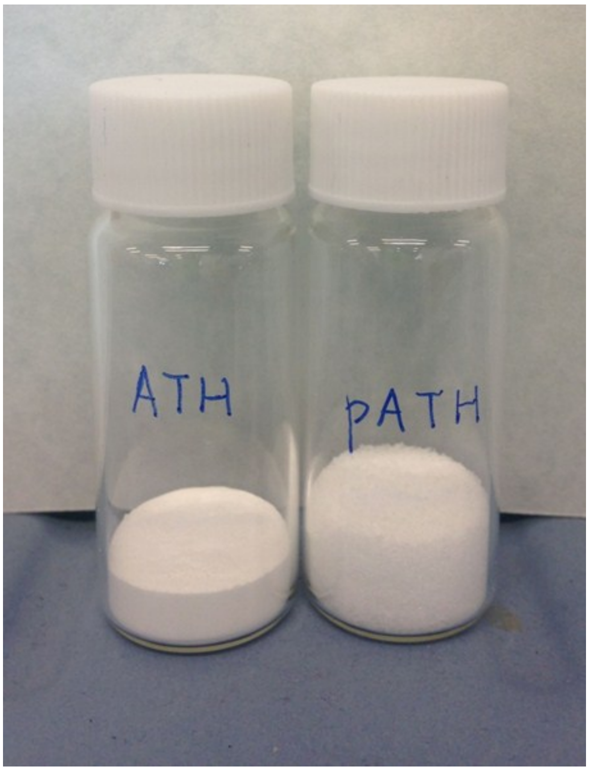


**Fig. S7** Digital photos of commercial ATH and pATH with same weight.

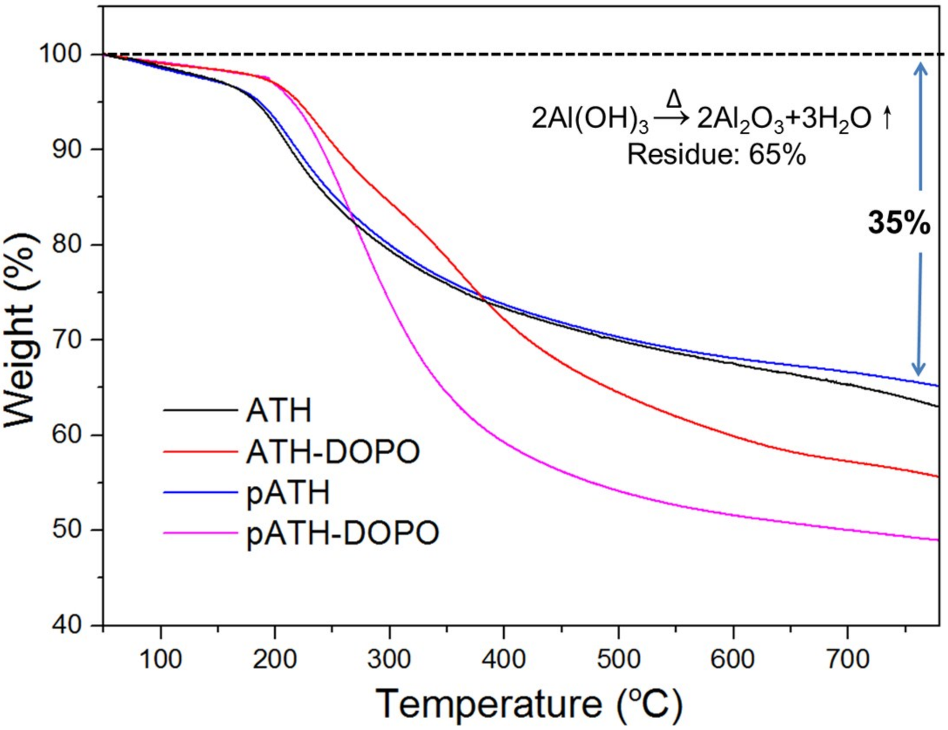


**Fig. S8** TGA profiles of ATH, ATH-DOPO, pATH and pATH-DOPO.

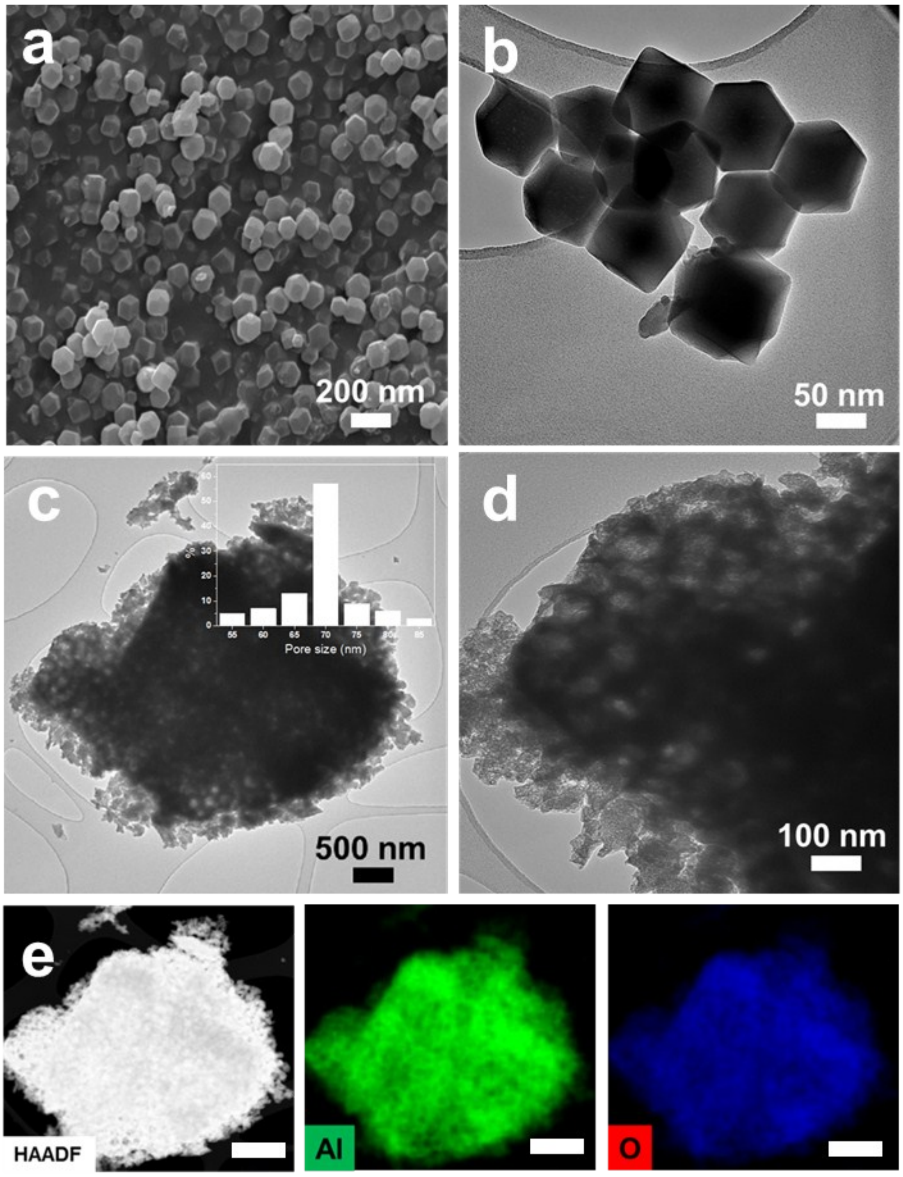


**Fig. S9** (a) SEM image of ZIF-8-5h; (b) TEM image of ZIF-8-5h; (c, d) TEM images of macro-pATH (The inset showed the pore size distribution); (e) HAADF image and elemental mapping of macro-pATH.

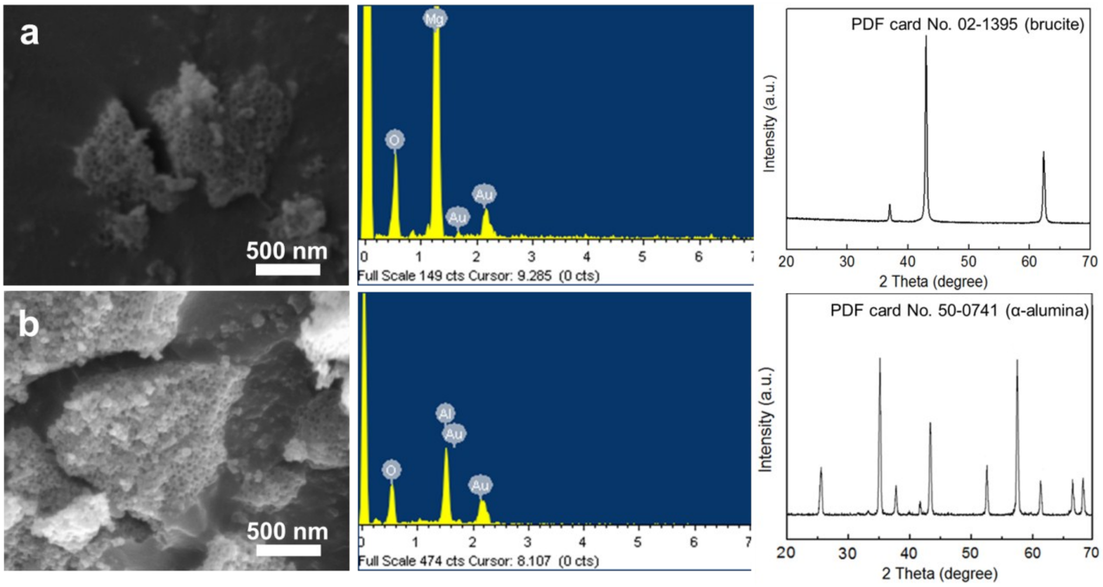


**Fig. S10** SEM images, EDX spectra and XRD patterns of (a) alumina and (b) magnesium hydroxide.

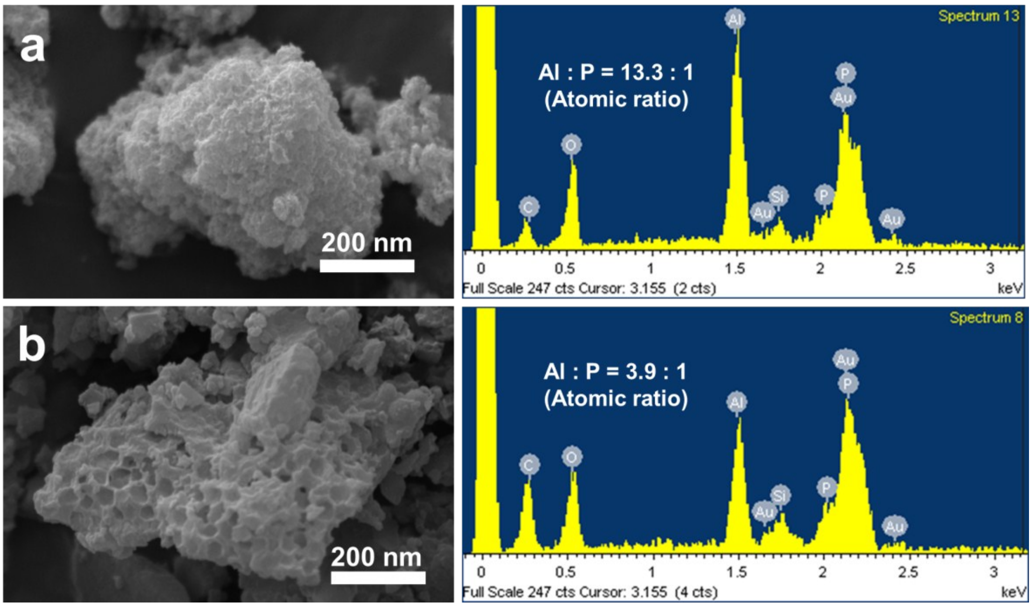


**Fig. S11** (a) SEM image and EDX spectra of ATH-DOPO; (b) SEM image and EDX spectra of pATH-DOPO.
